# Involvement of Oxidative Stress and Antioxidants in Modification of Cardiac Dysfunction Due to Ischemia–Reperfusion Injury

**DOI:** 10.3390/antiox14030340

**Published:** 2025-03-14

**Authors:** Naranjan S. Dhalla, Petr Ostadal, Paramjit S. Tappia

**Affiliations:** 1 St. Boniface Hospital Albrechtsen Research Centre, Institute of Cardiovascular Sciences, Department of Physiology & Pathophysiology, Max Rady College of Medicine, University of Manitoba, Winnipeg, MB R2H 2A6, Canada; 2 Department of Cardiology, 2nd Faculty of Medicine, Charles University, Motol University Hospital, V Uvalu 84, 15000 Prague, Czech Republic; ostadal.petr@gmail.com; 3 Asper Clinical Research Institute, St. Boniface Hospital, Winnipeg, MB R2H 2A6, Canada; ptappia@sbrc.ca

**Keywords:** ischemia–reperfusion injury, cardiac dysfunction, oxidative stress, antioxidants, subcellular defects, Ca^2+^-handling abnormalities

## Abstract

Delayed reperfusion of the ischemic heart (I/R) is known to impair the recovery of cardiac function and produce a wide variety of myocardial defects, including ultrastructural damage, metabolic alterations, subcellular Ca^2+^-handling abnormalities, activation of proteases, and changes in cardiac gene expression. Although I/R injury has been reported to induce the formation of reactive oxygen species (ROS), inflammation, and intracellular Ca^2+^ overload, the generation of oxidative stress is considered to play a critical role in the development of cardiac dysfunction. Increases in the production of superoxide, hydroxyl radicals, and oxidants, such as hydrogen peroxide and hypochlorous acid, occur in hearts subjected to I/R injury. In fact, mitochondria are a major source of the excessive production of ROS in I/R hearts due to impairment in the electron transport system as well as activation of xanthine oxidase and NADPH oxidase. Nitric oxide synthase, mainly present in the endothelium, is also activated due to I/R injury, leading to the production of nitric oxide, which, upon combination with superoxide radicals, generates nitrosative stress. Alterations in cardiac function, sarcolemma, sarcoplasmic reticulum Ca^2+^-handling activities, mitochondrial oxidative phosphorylation, and protease activation due to I/R injury are simulated upon exposing the heart to the oxyradical-generating system (xanthine plus xanthine oxidase) or H_2_O_2_. On the other hand, the activation of endogenous antioxidants such as superoxide dismutase, catalase, glutathione peroxidase, and the concentration of a transcription factor (Nrf2), which modulates the expression of various endogenous antioxidants, is depressed due to I/R injury in hearts. Furthermore, pretreatment of hearts with antioxidants such as catalase plus superoxide dismutase, N-acetylcysteine, and mercaptopropionylglycerine has been observed to attenuate I/R-induced subcellular Ca^2+^ handling and changes in Ca^2+^-regulatory activities; additionally, it has been found to depress protease activation and improve the recovery of cardiac function. These observations indicate that oxidative stress is intimately involved in the pathological effects of I/R injury and different antioxidants attenuate I/R-induced subcellular alterations and improve the recovery of cardiac function. Thus, we are faced with the task of developing safe and effective antioxidants as well as agents for upregulating the expression of endogenous antioxidants for the therapy of I/R injury.

## 1. Introduction

It is now well known that myocardial ischemia induces marked alterations in contractile function, cellular metabolism, and cardiac ultrastructure due to the lack of both oxygen and substrates. Although the reperfusion of the ischemic myocardium is beneficial in improving cardiac function and myocardial changes, delayed reperfusion, after a certain critical time of ischemic insult, has been shown to impair the recovery of cardiac function and exacerbate metabolic alterations, induce Ca^2+^-handling abnormalities, and promote damage to the myocardial structure [1,2,3,4,5,6,7,8,9,10,11,12,13,14,15]. The effects of delayed reperfusion are superimposed upon those for myocardial ischemia and are termed as ischemia–reperfusion (I/R) injury. Although the nature of defects due to I/R injury is not fully understood, this condition is generally associated with several adverse events such as arrhythmias, diverse signal transduction defects, mitochondrial dysfunction, microvascular damage, and cellular death. Furthermore, I/R injury is invariably seen in clinical conditions including acute coronary syndrome, angioplasty, thrombolysis, coronary-bypass surgery, cardiac transplantation, and stroke [16,17,18,19,20,21,22,23,24,25,26,27,28,29,30,31,32,33,34,35]. It should be mentioned that I/R injury is a complex health hazard, and the mechanisms of I/R-induced cardiac dysfunction are similar to those for hypoxia reoxygenation.

Cardiac dysfunction in hearts subjected to I/R injury has also been shown to involve oxidative stress, inflammation, endoplasmic reticulum stress, intracellular Ca^2+^-overload, and defects in subcellular organelles such as sarcolemma (SL), sarcoplasmic reticulum (SR), mitochondria (MT), and myofibrils (MF) [36,37,38,39,40,41,42,43,44,45,46,47,48,49,50,51]. However, oxidative stress is considered to play a critical role in the development of impaired cardiac performance due to I/R injury, because several defects associated with this pathological condition are elicited as its consequence. Furthermore, different antioxidants have been reported to attenuate the I/R-induced alterations in the heart [7,11,15,36,41,44,48,50,51,52]. It is therefore the objective of this article to provide an updated comprehensive review of various sources for the generation of oxidative stress as well as the role of endogenous antioxidants during the development of I/R injury. It is also planned to discuss the implications of oxidative stress in inducing cardiac dysfunction due to I/R injury. Furthermore, this study was undertaken with the intention of including evidence for the involvement of oxidative stress in I/R-induced subcellular alterations, particularly with respect to Ca^2+^-handling defects and the subsequent impairment of cardiac performance. In addition, the pharmacotherapy of I/R injury with both endogenous and exogenous antioxidant systems will be described for improving cardiac function in pathological conditions. Accordingly, an appropriate literature search was conducted on MEDLINE via PubMed using the following search terms: cardiac ischemia–reperfusion injury, reactive oxygen species, oxidative stress, antioxidants, Ca^2+^ handling, intracellular Ca^2+^ overload, and combination thereof was undertaken and the articles quoted in this review were selected to provide support of our hypothesis.

## 2. Generation of Oxidative Stress and Status of Antioxidant Systems in I/R Hearts

Oxidative stress is associated with an increase in the production of reactive oxygen species (ROS) and/or a decrease in the activities of antioxidant defense systems due to I/R injury in the heart [8,9,14,15,43,44,51]. There are five major types of ROS (superoxide radicals, hydroxyl radicals, hydrogen peroxide (H_2_O_2_), hypochlorous acid, and peroxynitrite) which are increased in the heart upon the induction of I/R injury. ROS are mainly generated by the impairment of electron transport in mitochondria, xanthine (X) + xanthine oxidase (XO) reaction, and arachidonic acid metabolism, as well as by the activation of monoamine oxidase, NADPH oxidase, the endothelium, and neutrophils. It should be mentioned that superoxide radicals are rapidly converted into H_2_O_2_, which is a precursor of hydroxyl ions in the presence of iron and copper, as well as hypochlorous acid (HOCl) in the presence of myeloperoxidases and chloride ions. Superoxide radicals also react with nitric oxide (produced upon the activation of endothelium) to form peroxynitrite. Although all members of the ROS family are inter-related, hydroxyl radicals are considered to be the most reactive, with a very short half-life. It should also be pointed out that low concentrations of ROS produce beneficial effects on the heart by activating different redox-sensitive signal transduction pathways, whereas high concentrations of ROS (oxidative stress) produce harmful effects by reacting with cellular proteins, lipids, carbohydrates, and DNA. Thus, I/R injury is known to produce changes in membrane permeability, protein thiol group oxidation, and lipid peroxidation, which are considered to be mediated through the generation of oxidative stress.

Excessive generation of ROS by mitochondrial defects and other sources have been demonstrated to be associated with the activation of transient receptor potential melastatin 2 (TRPM2), the production of endoplasmic reticulum stress (ER stress), DNA damage, and the upregulation of NADPH oxidase, heme oxygenases, and cyclooxygenases [53,54,55,56,57,58,59,60,61,62,63,64,65,66]. ROS have also been shown to produce ventricular arrhythmias, alter the apelinergic system and ubiquitin–proteosome axis, and induce different types of cell death, such as necrosis, apoptosis, necroptosis, pyroptosis, and ferroptosis [67,68,69,70,71,72,73,74,75,76,77,78]. Different endogenous proteins such as perilipin, SESTRIN2, heat shock protein 22, and humanin, as well as exosomes and microRNAs with antioxidant properties, have been reported to protect against the harmful effects of ROS in the heart [79,80,81,82,83,84,85]. Various enzymes, including superoxide dismutase (SOD), catalase, and glutathione peroxidases, as well as vitamins such as vitamin E, vitamin A, and vitamin C, are known to serve as endogenous antioxidants [8,44,49,50,51]. These antioxidants are considered to act through mechanisms involved in either scavenging ROS or inhibiting the generation of ROS in the myocardium. The levels of these antioxidants are not only decreased upon the reperfusion of an ischemic heart; these interventions are also effective in attenuating I/R injury to the heart upon pretreatment. These observations support the view that the generation of ROS and the development of oxidative stress are intimately involved in the pathogenesis of I/R injury to the heart [8,49,50,51,86,87].

## 3. Mechanisms of I/R-Injury-Induced Subcellular Defects and Cardiac Dysfunction

The status of cardiac function is determined by the coordinated Ca^2+^-handling activities of different subcellular organelles, including SL, SR, and MT, as well as the Ca^2+^-regulated activity of MF [11,88,89,90]; thus, it has been suggested that abnormalities in subcellular function due to oxidative stress play an important role in the development of cardiac dysfunction as a consequence of I/R injury [8,41,51]. It should be pointed out that myocardial ischemia upon occluding the coronary arteries is associated with a lack of oxygen, the inability of MT to oxidize substrates, and the accumulation of hydrogen in cardiomyocytes; these alterations result in an increase in the intracellular concentration of Ca^2+^ upon the stimulation of SL Na^+^-H^+^ exchange and SL Na^+^-Ca^2+^ exchange systems. The increased levels of Ca^2+^ in the ischemic myocardium activate XO and produce ROS through a reaction with X. Furthermore, the inability of MT to oxidize substrate in the ischemic heart is known to depress oxidative phosphorylation, impair the MT electron transport system, generate ROS, and produce the cessation of contractile activity. All these changes that are attributable to myocardial ischemia are reversible upon reperfusion; however, delayed reperfusion results in marked defects in cardiomyocytes, myocardial interstitium, endothelium, and coronary vasculature for the induction of ultrastructural abnormalities, as well as the impairment of contractile function recovery as a consequence of ROS production [1,2,3,4,5,6,7,8,9,10,11,41,43,51]. Such alterations in cardiomyocytes due to reperfusion of the ischemic heart are associated with a further increase in the entry of Ca^2+^ due to an increase in membrane permeability as well as depressions in SL Na^+^-K^+^ ATPase and SL Na^+^- Ca^2+^ exchange activities. In addition, there occurs a depression in the SR Ca^2+^ pump activity and leakage of Ca^2+^ from the SR tubular system due to ROS-induced alterations in the SR membrane. These Ca^2+^-handling abnormalities in SL and SR lead to the development of intracellular Ca^2+^ overload and subsequent MT Ca^2+^ overload, which is known to induce further defects in the MT electron transport system, oxidative phosphorylation activity, and energy production; in addition, it leads to the generation of oxidative stress, the opening of MT pores, and the release of cytotoxic substances for the induction of programmed cell death.

Although oxidative stress is considered to be mainly generated by MT upon inducing I/R injury, the involvement of other systems cannot be overlooked. Examples of these include the following: Ca^2+^-handling abnormalities in SL and SR for the occurrence of MT Ca^2+^ overload; endothelium nitric oxide synthase for the production of nitric oxide; peroxynitrite, macrophages, and leukocytes for the release of proinflammatory cytokines such as tumor necrosis factor-α (TNF-α) and interleukins (IL-1β and IL-6); long noncoding RNA–RNA axis and thioredoxin-interacting protein for the generation of oxidative stress during the development of I/R injury [91,92,93,94,95,96,97,98,99]. Furthermore, the activation of both SL- and MT-associated NADPH oxidases and glucose homeostasis, as well as the oxidation of free fatty acids and arachidonic acid metabolic pathways, have been demonstrated to play a critical role in the development of oxidative stress during the initiation and progression of I/R injury to the heart [20,21,51,100]. It is also noteworthy that the concentrations of different ROS and oxidants are increased in the heart upon inducing I/R injury, whereas those for endogenous antioxidant enzymes are decreased [49,50,51,101,102,103]; these observations provide strong evidence for the occurrence of oxidative stress during the development of oxidative stress. Several in vivo studies have also shown that the activities of different transcription factors such as nuclear factor erythroid 2-related factor 2 (Nrf2) and Kelch-like ECH-associated protein 1 (Keap 1), which modulate the levels of various antioxidants, are depressed due to I/R injury [104,105,106,107,108,109,110,111,112]. Accordingly, the upregulation of both transcription factors (Nrf2 and Keap1) is considered to be an excellent strategy for reducing oxidative stress responses, subcellular Ca^2+^-handling defects, and the development of intracellular Ca^2+^ overload due to I/R injury.

## 4. Pharmacotherapy and Cardioprotection of I/R-Induced Injury

Extensive experimental work in the area of pharmacotherapy and cardioprotection has been carried out to investigate the beneficial effects of several interventions in preventing I/R injury to the heart [8,44,51,113]. Different pharmacologic agents such as hypoglycemic drugs [114,115], aldosterone inhibitors [116], regulatory interventions (including SIRT1 (regulator of autophagy), STING (stimulator of interferon genes), and AMP-activated protein kinase [117,118,119]), and nanomedicines [120] have been found to attenuate I/R injury. Likewise, some interventions, such as nitric oxide, heme oxygenase 1, propofal, and adiponectin (which affects diverse molecular targets), were observed to partially prevent I/R injury in the heart [121,122,123,124]. Furthermore, different types of RNAs (circular RNA, noncoding RNA, and microRNA) have also been reported to exert beneficial effects in attenuating I/R injury through some complex mechanisms [125,126,127]. Some phytochemicals, natural products, and micronutrients have been claimed to protect against I/R injury by improving antioxidant status [128,129,130]. It may well be the case that all the interventions indicated in this section partially depress I/R injury by reducing the formation of ROS or increasing the concentrations of different antioxidants; however, the involvement of other mechanisms, such as alterations in inflammation, reduction in intracellular Ca^2+^ overload, or the inhibition of protease activation, cannot be ruled out with any certainty.

In view of the depressed endogenous antioxidant levels in the I/R hearts, several investigators have used redox sensitive therapy for preventing I/R injury. In this regard, supplementation with exogenous antioxidant preparations [51,52,126,131,132] and the administration of interventions which upregulate the transcription factor Nrf2 [107,110,111], through increasing antioxidant status, have been shown to exert beneficial effects against I/R injury. Various proteins, such as humanin, berberine, sestrin, taurine, fisetin, quercetin, and polydatin, all with antioxidant properties, have also been observed to attenuate I/R injury in the heart [81,122,133,134,135,136,137]. Different vitamins, such as vitamins A, C, and E, with antioxidant activities, have also been shown to exert beneficial effects against I/R injury [138,139]. Some gases, such as molecular hydrogen and hydrogen sulfide [109,131,140,141,142,143], as well as ischemic preconditioning [90,144], have also been reported to exert beneficial actions. All these observations, showing cardioprotective effects of various interventions in I/R hearts due to their antioxidant activities, support the view that oxidative stress plays a pivotal role in the development of I/R injury.

## 5. Evidence for the Involvement of Oxidative Stress in I/R-Induced Cardiac Dysfunction

The intention here is to provide further evidence for the occurrence of oxidative stress in the impairment of cardiac function recovery upon the reperfusion of the ischemic heart. Accordingly, we have analyzed some information from some experiments which were carried out by inducing I/R injury in isolated perfused rat hearts that were pretreated in the absence and presence of some antioxidants. It should be mentioned that isolated heats were subjected to 30 min of global ischemia before inducing reperfusion for 30 to 60 min. Furthermore, the effects of I/R-induced injury were compared with those obtained from rat hearts perfused with or without oxyradical-generating system (X plus XO) as well as H_2_O_2_, a well-known oxidant, for 30 min. We have shown that the reperfusion of the ischemic heart results in a depression of the left ventricular developed pressure, while the left ventricular end diastolic pressure is markedly increased, demonstrating that reperfusion of the ischemic heart impairs the recovery of cardiac function [87]. We have further reported an increase in the biomarkers of oxidative stress (H_2_O_2_ content and malondialdehyde) in hearts subjected to I/R [87]. Treatment of hearts with an antioxidant mixture (SOD–catalase) was observed to attenuate the I/R-induced changes in cardiac function and biomarkers of oxidative stress [87]. In addition, the myocardial Ca^2+^ contents in control, I/R, and SOD–catalase-treated I/R hearts were reported to be 8.4 ± 1.2, 22.6 ± 2.9, and 9.8 ± 1.6 µmol/g dry wt., respectively [87]. These data revealed the impact of oxidative stress and intracellular Ca^2+^ overload and the beneficial actions of antioxidants on cardiac function.

The I/R-induced myocardial functional changes were demonstrated to be associated with a marked reduction in SL Na^+^-K^+^ ATPase activity as well as an increase in matrix metalloproteinase (MMP) and calpain activities upon the reperfusion of ischemic hearts [145]. The protective role of antioxidants was further demonstrated by the attenuation of these I/R-induced effects when the reperfusion of ischemic hearts was carried out in the presence of well-known antioxidants, namely N-acetylcysteine (NAC) or mercaptopropionyl glycine (MPG) [145]. On the other hand, the perfusion of hearts with X plus XO was also shown to be associated with reduced Na^+^-K^+^ ATPase activities as well as increased activities of both MMP and calpain. Other subcellular activities, including SL Na^+^-Ca^2+^ exchange, ATP-dependent Ca^2+^ uptake, and Ca^2+^-stimulated ATP activities were depressed upon reperfusing ischemic hearts or when hearts are perfused with a medium containing X plus XO. These changes in the SL enzyme or Ca^2+^-handling activities in I/R hearts or in hearts exposed to an oxyradical-generating system were shown to be partially normalized in the presence of SOD–catalase [146,147,148].

It was also shown that, while SR Ca^2+^ uptake, Ca^2+^-stimulated ATPase, Ca^2+^ release, and ryanodine-binding activities were depressed in I/R hearts, the reperfusion of ischemic hearts in the presence of SOD–catalase attenuate these alterations (except that for Ca^2+^-stimulated ATPase) [149]. Furthermore, the perfusion of hearts with X plus XO or H_2_O_2_ [149] followed by reperfusion also depressed SR Ca^2+^ uptake, Ca^2+^-stimulated ATPase, Ca^2+^ release, and ryanodine binding activities [149]. Taken together, these observations indicated that defects in both SL and SR organelles due to I/R injury can not only be prevented by antioxidant mixtures, but are simulated when hearts are exposed to ROS-generating systems.

The effects of I/R injury and ROS-generating systems on other subcellular organelles, such as mitochondria (MT) and myofibrils (MF), have also been examined. In this regard, depressions in MT state 3 respiration and ADP/O ratio index were reported upon subjection of the heart to I/R injury; these were attenuated by SOD–catalase [150]. Similarly, perfusion of the rat heart with X+XO or H_2_O_2_ [150] also reduces MT state 3 respiration and the ADP/O ratio index. In addition, the I/R-induced depression observed for MF Ca^2+^-stimulated ATPase activity was prevented in the presence of SOD–catalase or N-acetylcysteine [151,152]. It was also noted that MF Mg^2+^-ATPase activity was increased when perfusing the isolated rat hearts with X+XO or H_2_O_2_, whereas MF Ca^2+^-stimulated ATPase activity was depressed. Although the exact reason for the difference in the responses of MF Mg^2+^-ATPase to I/R injury and oxidative stress is not clear, MF Ca^2+^-stimulated ATPase activity was depressed under both experimental conditions. These observations suggest that both I/R injury and the ROS-generating system produce similar changes in the MT and MF and the effects of I/R injury were attenuated by antioxidants.

In view of the critical role of SR Ca^2+^ handling as well as the involvement of oxidative stress in the development of intracellular Ca^2+^ overload upon the induction of I/I injury, the effects of NAC and MPG on I/R-induced and X–XO-induced changes in SR Ca^2+^ uptake and SR Ca^2+^ release activities were examined in isolated rat heart [48]. In this regard, I/R-induced depressions in Ca^2+^ uptake and SR Ca^2+^ release activities were attenuated by both NAC and MPG treatments. Likewise, treatments of hearts with NAC or MPG mitigated the X–XO-induced alterations in cardiac Ca^2+^ uptake and SR Ca^2+^ release activities [48]. These data, with exogenous antioxidants (NAC and MPG), support observations regarding the beneficial effects of endogenous antioxidant mixtures (SOD plus CAT) on I/R-induced decreases in SR Ca^2+^ transport activities.

**Figure 1:**
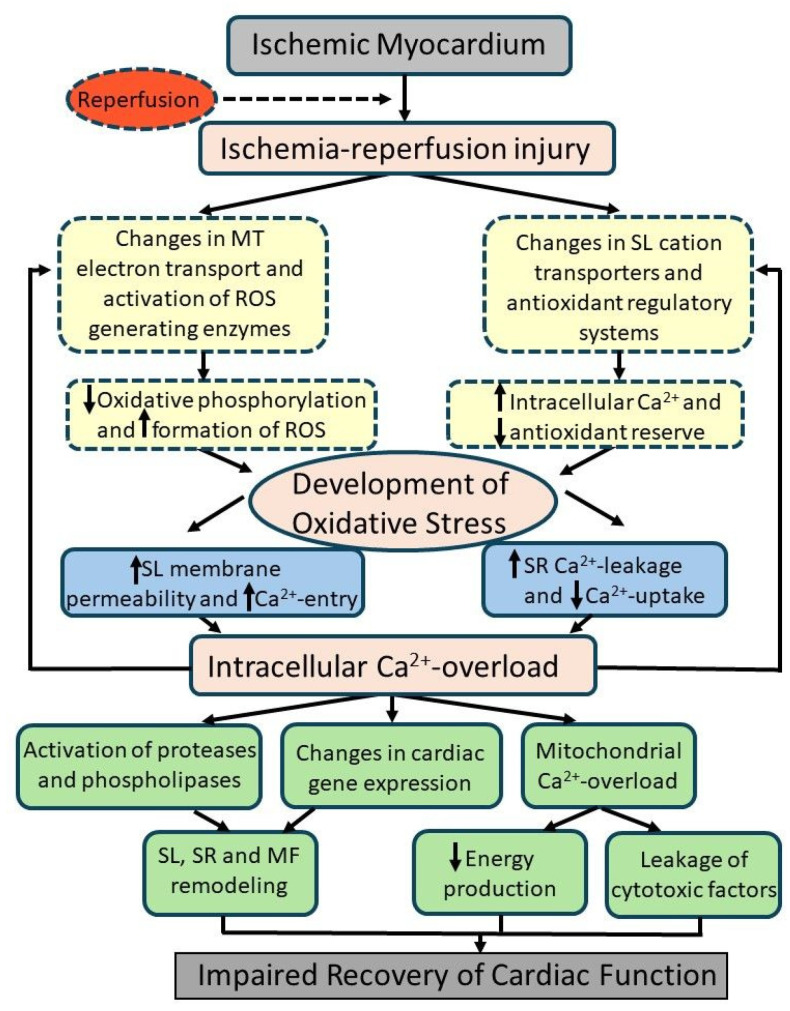
Schematic representation of reperfusion-induced events in ischemic myocardium (ischemia–reperfusion injury) involving subcellular alterations for the generation of oxidative stress, development of intracellular Ca^2+^ overload, and impaired recovery of cardiac function. MT = mitochondria, SL = sarcolemma; ROS = reactive oxygen species; SR = sarcoplasmic reticulum; Ca^2+^ = calcium; MF = myofibrils.

Although the role of oxidative stress and intracellular Ca^2+^ overload in I/R injury has been demonstrated in experimental studies by employing different animal models, the beneficial effects of various antioxidants in the clinical setting remain to be conclusively established [48,51,87,153]. Nonetheless, the lower sensitivity of female hearts to I/R injury in comparison to male hearts has been attributed to the reduction in Ca^2+^ handling as well as oxidative stress [154,155,156,157,158,159,160]. A schematic representation of the complex relationship involved in the development of oxidative stress and intracellular Ca^2+^ overload, as well as the occurrence of subcellular Ca^2+^-handling abnormalities, is depicted in Figure 1. It should be mentioned that the mechanisms for the reduction of antioxidant levels in I/R injury are not understood, whereas the depression in transcription factors such as Nrf2 under in vivo conditions may be involved in reducing endogenous antioxidant levels and activities.

It can be argued that we have provided the above evidence in support of the involvement of oxidative stress and the beneficial effects of antioxidants on I/R injury from the data obtained in our laboratory. However, it is pointed out that there is a real lack of information from other laboratories on I/R-induced alterations in subcellular organelles and Ca^2+^-handling activities that can be attributed to oxidative stress; thus, it is essential to emphasize the involvement of the interaction of oxidative stress, antioxidants, and subcellular Ca^2+^-handling activities. Nonetheless, there is a wealth of information regarding the role of oxidative stress in I/R-induced changes in cardiac function [161,162,163,164,165,166,167] as well as structural factors [168,169,170,171,172,173,174], metabolic factors [175,176,177], apoptosis [178,179,180], signal transduction [32,181,182,183,184,185], and arrhythmias [186,187,188,189]. These observations support our viewpoint indirectly.

## 6. Perspectives and Conclusions

From the foregoing discussion, it is evident that both oxidative stress and intracellular Ca^2+^ overload induce I/R injury to the heart. Since both these pathogenic factors are inter-related, it is difficult to determine the sequence of their development as well as their cause–effect relationship for inducing I/R injury. In this regard, it should be mentioned that changes occur in the MT electron transport system and in energy production due to myocardial ischemia; moreover, a large amount of H_2_ is accumulated in the ischemic myocardium. Reperfusion of the ischemic heart removes H_2_ from cardiomyocytes upon stimulating the SL Na^+^-H^+^ and SL Na^+^-Ca^2+^ exchange systems, which increases the intracellular concentration of Ca^2+^ and subsequently activates different proteases and phospholipases. These changes will depress SR Ca^2+^ pump activity to further promote the elevation of intracellular Ca^2+^. In addition, the increased protease and phospholipase activities will increase the permeability of both SL and SR mechanisms for increasing the entry of extracellular Ca^2+^ and leakage of Ca^2+^ from SR stores, respectively. Such defects in Ca^2+^ handling by SL and SR will result in the occurrence of intracellular Ca^2+^ overload and marked accumulation of Ca^2+^ in MT. Thus, MT Ca^2+^ overload will be associated with the generation of ROS due to further derangement of the electron transport system as well as activation of different enzymes, including XO and NADPH oxidase, leading to the generation of ROS. This excessive production of ROS will result in the occurrence of oxidative stress, the intensity of which will be further promoted by a depression in the level of endogenous antioxidants.

It is now well known that the delayed reperfusion of the ischemic heart produces marked alterations in myocardial metabolism and cardiac ultrastructure in addition to impairing the recovery of cardiac function. Such changes in I/R hearts are associated with the increased formation of ROS. It is noteworthy that MT are the major source of ROS production due to defects in the electron transport system; another major source is the activation of XO and NADPH oxidase, as a consequence of reperfusion of ischemic hearts. The activation of endothelium nitric oxide synthase and the formation of nitric oxide also occur; these combine with superoxide radicals to generate peroxynitrite. On the other hand, the concentrations of endogenous antioxidants, including superoxide dismutase, catalase, and glutathione peroxidase (which antagonizes the actions of ROS), are decreased in I/R hearts. It should also be mentioned that, under in vivo conditions, the levels of transcription factors such as Nrf2, which regulate the status of several antioxidants, are also depressed upon reperfusion. Thus, an imbalance occurs between the excessive formation of ROS and the reduced levels of antioxidants due to reperfusion; these lead to the generation of oxidative stress. Although various defects occur in the I/R hearts, such as inflammation, subcellular alterations, intracellular Ca^2+^ overload, activation of proteases, and changes in cardiac gene expression, these abnormalities seem to be the consequence of oxidative stress generation during the development of I/R injury.

Impaired recovery of cardiac function due to I/R injury has been shown to be associated with depression in SL Na^+^-K^+^ ATPase, Na^+^-Ca^2+^ exchange, and ATP-dependent Ca^2+^ pump activities. Furthermore, I/R injury has been demonstrated to induce changes in the SR Ca^2+^ pump activity and promote the leakage of Ca^2+^ to further raise the intracellular concentration of Ca^2+^ and cause the development of MT Ca^2+^ overload, depression in the oxidative phosphorylation, and energy production, opening MT pores for the leakage of cytotoxic material and the development of programmed cellular death. The occurrence of Ca^2+^-handling abnormalities in the SL and SR organelles in I/R hearts as a consequence of oxidative stress is also associated with the development of intracellular Ca^2+^ overload, the activation of proteases such as MMP and calpain, depression in the cardiac gene expression, and the loss of MF Ca^2+^ sensitivity. Furthermore, treatment of the heart with different antioxidants has been shown to attenuate I/R-induced Ca^2+^-handling defects in subcellular organelles, activation of proteases, loss of MF Ca^2+^ sensitivity, and development of cardiac dysfunction. It is thus evident that both oxidative stress and subcellular Ca^2+^-handling defects play important roles in impairing the recovery of cardiac function due to I/R injury. Accordingly, it is suggested that efforts should be made to develop safe and effective interventions for the upregulation of endogenous antioxidants for improving cardiovascular abnormalities associated with I/R injury.

## Data Availability

The original contributions presented in this study are included in the article.
